# Development of Meat Products with Healthier Lipid Content: Vibrational Spectroscopy

**DOI:** 10.3390/foods10020341

**Published:** 2021-02-05

**Authors:** Claudia Ruiz-Capillas, Ana M. Herrero

**Affiliations:** Institute of Food Science, Technology and Nutrition (ICTAN-CSIC), José Antonio Novais 10, 28040 Madrid, Spain; claudia@ictan.csic.es

**Keywords:** healthier meat products, lipid content, structured lipids, emulsion gels, oil bulking agent, vibrational spectroscopy, Raman spectroscopy, infrared spectroscopy, technological properties

## Abstract

This review focuses on the importance of developing meat products with healthier lipid content and strategies such as the use of structured lipids to develop these enriched products. The review also conducts a critical analysis of the use of vibrational spectroscopy as a tool to further these developments. Meat and meat products are extensively recognized and consumed in the world. They are an important nutritional contribution in our diet. However, their consumption has also been associated with some negative consequences for health due to some of its components. There are new trends in the design of healthy meat products focusing mainly on improving their composition. From among the different strategies, improving lipid content is the one that has received the most attention. A novel development is the formation of lipid materials based on structured lipids such emulsion gels (EGs) or oil-bulking agents (OBAs) that offer attractive applications in the reformulation of health-enhanced meat products. A deeper interpretation is required of the complicated relationship between the structure of their components and their properties in order to obtain structured lipids and healthier meat products with improved lipid content and acceptable characteristics. To this end, vibrational spectroscopy techniques (Raman and infrared spectroscopy) have been demonstrated to be suitable in the elucidation of the structural characteristics of lipid materials based on structured lipids (EGs or OBAs) and the corresponding reformulated health-enhanced meat products into which these fat replacers have been incorporated. Future research on these structures and how they correlate to certain technological properties could help in selecting the best lipid material to achieve specific technological properties in healthier meat products with improved lipid content.

## 1. Introduction

The global dynamics of the production and consumption of meat and meat products has evolved rapidly as the result of changing lifestyles and nutritional ideologies among part of the population. As a result, it is important to address several different aspects regarding the quality of meat and meat products, particularly nutritional (as relates to health), safety and sustainability aspects.

Regarding the nutritional aspects of meat and meat products, different strategies have been explored to optimize the composition of these products to make them healthier and to bring them in line with health recommendations and nutritional guidelines promoted by public bodies in response to new consumer demands.

Of the different strategies to improve meat and meat product composition, lipid content optimization has attracted the most attention owing to health recommendations [1]. This typically entails the partial or nearly complete substitution of fat with other healthier lipids by means of different technological procedures [1]. However, the most appropriate procedure in each case depends on the type of product, lipid material used, and the nature and magnitude of the proposed change. In the last few years, there has been growing attention in developing lipid materials to reduce or even completely replace fat in order to develop health-enhanced meat products. However, in most cases it is difficult to completely replace fat in food products. The physical and thermal properties of these products should be similar to those of animal fat but with fewer calories and an improved lipid profile [2,3]. A recent novel development is the formation and use of structured lipids such as emulsion gels (EGs) or oil-bulking agents (OBA) [4,5] that provide remarkable applications in the reformulation of health-enhanced meat products [2,6,7,8,9,10]. Although in some isolated cases, oil-structuring procedures applied to the reformulation of meat products as animal fat replacers could affect some of the properties of the final product, they have been successful in creating food that is viable in terms of technology, microbiology, sensorial properties and safety [2,3,6,7,9], with a shelf life similar to that of its standard meat counterpart. These animal fat replacers can improve the technological properties of meat products (water and fat content), and can also be employed as carriers of different nutrients (fiber, minerals, phenolic compounds, etc.), some with biological activity offering health benefits [6,7,9,11,12].

Successful development of structured lipid materials with healthier characteristics requires in-depth study of their nutritional, sensorial and technological characteristics, and a broader understanding of the complex relationship between the structure of their components and their properties. A new approach to the understanding of these interrelationships based on an analysis of the conformational modifications taking place within the components of lipid material and reformulated new meat products that affect their properties, merits consideration. In these connections, vibrational spectroscopic techniques such as Raman and infrared spectroscopy are direct and non-invasive techniques which offer an extensive range of possibilities in meat and meat products [6,7,13] and show great potential in supplementing structural information relating to fat-replacer production processes and the reformulation of health-enhanced meat products into which they are ultimately incorporated [4,5,6,7]. These techniques are also useful for quality control as it has been shown that their structure correlates with traditional ways of assessing meat quality (water retention capacity, texture, etc.) [13]

Based on the above, this review features a brief description of the novel strategies used to develop meat products with a healthier lipid content based mainly on the use of structured lipids (emulsion gels and oil bulking agents). It also proposes the employment of vibrational spectroscopy (infrared and Raman spectroscopy) to enhance the development of both structured lipids and the meat products which are included as animal fat substitutes. Moreover, the basic concepts of infrared and Raman spectroscopy are reviewed to aid in the understanding of how these spectroscopic techniques are applied.

## 2. Meat and Meat Products with a Healthier Lipid Content

Meat and meat products form part of the diet of many consumers in the world and are a significant source of an extensive range of nutrients essential for healthy development. These foods provide many nutrients such as high-value protein, fatty acids such as conjugated linoleic acid, minerals (iron, zinc and selenium) and B-complex vitamins. However, meat products also contain processing additives (sodium, nitrites, high fat content, etc.) with negative health implications. This has sparked the development of meat products more in line with health recommendations [14] using different procedures to enhance the composition of meat and especially meat products [1,2,3,15]. These procedures are mostly based on animal production (genetics and nutrition) and/or technological procedures focused on reducing or minimizing compounds with negative health implications and increasing compounds with positive health implications [1,2,3]. From among the technological strategies which aim to improve the global composition of meat products, special mention should be made to meat product reformulation processes which aim to remove, reduce, increase, add and/or replace certain components to develop healthier meat products [1,2,3,15].

From among the different reformulation processes, improved lipid content (in qualitative (lipid profile) and quantitative terms) is one of the most relevant processes in the development of healthier meat products [1,2,3,15] because there is an increasing indication of the connection between dietary fat intake and chronic disorders such as ischemic heart disease, some forms of cancer, obesity, and other ailments [14,16,17,18]. More specifically, some studies have shown a correlation between the intake of saturated fatty acids (SFAs) and an increase in total cholesterol levels in the blood. An increase in High-density lipoprotein (HDL) (ratio HDL/total cholesterol) is generally considered to be positive, whereas an increase in Low-density lipoprotein (LDL) is detrimental to health.

The consumption of SFA has been related to the development of cardiovascular disease, obesity, hypertension and some types of cancer [19]. The consumption of monounsaturated fatty acids (MUFAs), among which oleic acid stands out due to its high prevalence, has also been related to a reduction in LDL cholesterol and triglycerides in the blood. However, the role played by SFAs and MUFAs in human health is a controversial topic. Other studies question the health effects of SFAs and certain unsaturated fatty acids [20,21].

Despite this controversy, general consumption recommendations of total and unsaturated fatty acids can be made [14,22]. Briefly, dietary fat intake should preferably account for between 20–35% of total daily calories consumed [14,22]. According to dietary recommendations for the intake of specific fatty acids as a proportion of total diet, no more than 10% of calorie intake should be from SFAs, 6–10% from poly-unsaturated fatty acids (PUFAs) (n-6 and n-3, 6–11%), between 16–19% from MUFAs, and less than 1% from total fatty acids (TFAs) [14,22].

To meet these recommendations and improve the lipid content of meat products, technological strategies generally replace animal fat with different lipids, mainly from plant and marine sources, more in line with health recommendations (e.g., lower proportion of saturated fatty acids (SFA), higher in monounsaturated (MUFA) and polyunsaturated fatty acids (PUFA), especially from the omega-3 or n-3 family of fats). Efforts are also being made to improve PUFA content, the n-6/n-3 and PUFA/SFA ratios and, where possible, to reduce cholesterol and trans-fatty acids) [1]. This will predictably reduce the risk of developing the diseases discussed above.

**Figure 1:**
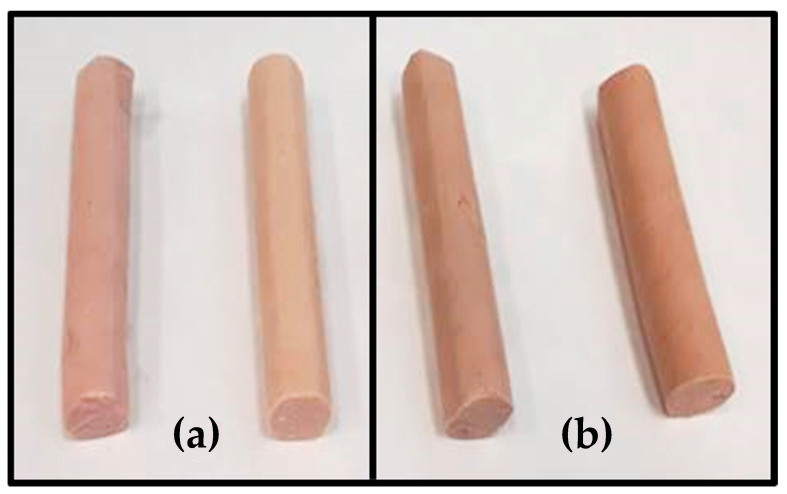
Example of healthy meat products with enhanced lipid content based on used structured lipids (as an animal fat replacer: (**a**) emulsion gels) and (**b**) oil bulking agent.

Different procedures have been used to substitute animal fat with healthier lipids, ranging from the most conventional (direct addition, encapsulated, emulsified form, etc.) to the most novel recently developed techniques such as lipid structuring with EGs or the oil-bulking agents referred to above [1,2,3]. To that end, different vegetable oils (olive, soybean, etc.), marine oils (fish and algae), or mixtures of these have been employed to partially or completely substitute animal fat in various types of meat derivates (fresh, cooked, and dry cured). However, studies have shown that meat products reformulated in this way have different physicochemical characteristics which could impact negatively on the preferred quality parameters of the reformulated product [1,2,3]. Nevertheless, novel strategies such as lipid restructuring can be employed to enhance the quality of the reformulated meat products since these lipid materials and generate a solid fat which maintains solid-like properties. This could be a better way to develop meat products with a healthy lipid profile without negatively impacting their characteristics (Figure 1).

### Lipid Materials Based on Structured Lipid: Emulsion Gels and Oil Bulking Agents

Lipid materials created using lipid structuring procedures have obtained a great deal of attention in the context of meat products. These lipid constituents can be employed as fat substitutes in meat products thanks to their solid-like properties simulating animal fat [1,2,3]. Their characteristics are of great interest for their application in meat products from a nutritional (healthy composition) and technological point of view. Of the various types of structured lipids EGs and OBAs are the most interesting due to their singular properties and possibilities as animal fat substitutes in meat products [1,2,3].

EGs are lipid materials in which emulsions and gels (hydrogels) concur. Formulation of these EGs fundamentally includes a lipid phase (olive, microalgal and chia oils, among others), an aqueous phase, an emulsifier (soy and whey protein, among others) and a gelling process [1,2,3]. Different compounds can be used in their formulation to give them particular technological characteristics, comprising specific bioactive compounds that provide nutritional benefits [2]. There are various procedures that can be used to create the gelling process such as heating, acidification, addition of divalent cations (Ca^2+^), and enzymes with hydrocolloids [15,23]. Particular attention is being paid to cold gelling strategies employing binding agents such as alginates which form cold-set EGs [2,4].

Other interesting structured lipids are OBAs based on the dispersion of oil droplets in a continuous aqueous matrix-forming gel [2]. In these OBAs, liquid oil such as olive oil is usually enclosed in a hydrogel network structure. The formation of these OBAs includes mostly an early phase where the oil is distributed in the aqueous phase and lastly the gelation of the aqueous phase is induced by a gelling agent such as alginate. This procedure provides the OBAs with a solid structure, permitting it to be incorporate, for example, as an optimal fat replacer in meat products. Different gelling agents such as hydrocolloids (alginate) have been employed independently or in a mixture, causing diverse textures and structures in the final OBA [15].

Many studies have been conducted in recent years concentrating on the creation and characterization of EGs and their application in food products (yoghurt, cheese, sauces), particularly in the elaboration of cooked and fresh meat products (frankfurter type cooked sausages, fresh sausages, hamburgers, etc.) with a healthier lipid content [2,15,24,25]. However, OBAs have not been extensively employed in food science but their application as animal fat substitutes in healthier meat products is especially remarkable. These works have been focused mainly in the composition and technological characterization of EGS and OBAs. However, a further study of their structure and of the interactions between their diverse components, and their technological characterization, is required to gain greater insight into the different essential aspects necessary for their practical application as animal fat substitutes in meat products for the purpose of creating health-enhanced meat products. We likewise require a better interpretation of the changes in the structure of the main components found in meat products (proteins, lipids, etc.) that take place when structured lipids are incorporated as animal fat replacers, and how these affect the end product’s technological properties. This knowledge will help to improve and optimize the development of health-enhanced meat products with specific technological characteristics. A useful approach could be to use vibrational spectroscopy (infrared and Raman spectroscopy) to analyze structural modifications in proteins and lipids in the formation of lipid materials and in the health-enhanced meat products in which these materials are incorporated as animal fat replacers.

To better understand the use of vibrational spectroscopy in the development of EGs and OBAs and in meat products with a healthier lipid content, it is necessary to adequately know the basic concepts of these techniques and the interpretation of the spectral results obtained with them.

## 3. Basic Concepts of Vibrational Spectroscopic Techniques: Infrared and Raman Spectroscopy

Vibrational spectroscopy, which involves infrared (IR) and Raman spectroscopies, is founded on the transitions concerning quantized vibrational energy states of molecules. In IR spectroscopy, the energy for these transitions is supplied by radiation in the IR regions (mid-IR and near-infrared) of the electromagnetic spectrum [26]. In Raman spectroscopy, samples are excited with monochromatic incident radiation that may be in the ultraviolet (UV), visible (VIS) or near-infrared (NIR) regions of the electromagnetic spectrum [26]. Complementary data on fundamental vibrational modes can be found from mid-IR and Raman spectra, as some vibrational motions are perceived mainly with IR radiation and others mostly by Raman scattering. In relation to protein structure, both vibrational spectroscopic techniques provide information about the secondary structure of proteins (α-helix, β-sheet, unordered), but Raman spectroscopy can also provide more detailed information about the tertiary structure of proteins [13,27,28,29]. Regarding structural changes in lipids, both provide relevant information (particularly in meat products with improved lipid content) and both have been used to provide information about the changes in their lipid structure. However, it should be noted that IR is faster and requires a smaller sample size than Raman spectroscopy [13,27,28,29].

IR and Raman spectroscopy provide many special benefits in food researches [13,27,28,29]. These techniques can be used to condensed-phase samples in several physical states, whether liquid or solid, clear or opaque. In many situations, insignificant or no sample pre-treatment is needed, and a spectrum can habitually be obtained quite fast [13,27,28,29]. There has been a remarkable increase in mid-infrared applications resulting from the progress of mid-infrared Fourier transform (FT-IR) spectrometers in combination with sampling methods comprising attenuated total reflection (ATR), for solids, semisolids and liquids, due to the benefits they offer [13,27,28,29]. Additionally, FT-IR microscopy has paved the way for novel uses of in situ microspectroscopic food mapping and imaging. In the last few years, hyperspectral imaging (HSI) has appeared as a hopeful analytical method for quality control and has involved a lot of attention in the non-destructive analysis of food products. Similarly, Fourier transform Raman spectroscopy (FT-Raman), using NIR excitation from a Nd: YAG laser at 1064 nm, can usually solve the drawback of fluorescence in foods [29]. Methods such as surface-enhanced Raman spectroscopy (SERS), confocal Raman microspectroscopy, and Raman imaging spectroscopy are established for their possibilities and special benefits in examining food components at very low amounts, and for in situ multi-component determination [26,30].

### 3.1. Analysis of Infrared and Raman Spectra Data

Infrared and Raman spectroscopy offer relevant data on the structure of the components of meat and meat products (proteins, lipids, water, etc.) non-invasively and in situ [26,30]. The reformulation processes used to develop meat products with an improved lipid content can modify Raman and infrared spectral bands due to structural changes in meat components (proteins, lipids and water). Basic information is therefore needed about how these changes are analyzed separately in proteins and lipids. For qualitative analysis, modifications can be visualized by comparing the spectrum in question with the characteristic bands of proteins, lipids or water. This requires an analysis of changes in the intensity, frequency, and half-widths of the Raman and infrared bands of chemical groups of proteins, lipids and water which are indicative of qualitative structural changes [13,27,29]. For quantitative analysis, curve-fitting of these bands is often used [13,27,29]. All this structural information is briefly described in the following sections.

#### 3.1.1. Infrared Spectra

Mid-infrared (IR) spectroscopy is the most widely used spectroscopic technique in meat and meat products as it provides fundamental information about protein and lipid structure through well-resolved characteristic infrared bands. In recent years, mid-infrared Fourier transform (FT-IR) spectrometers with attenuated total reflection (ATR) stand out from among the different mid-infrared (IR) spectroscopy techniques employed to analyze the structure of meat and meat products.

**Figure 2:**
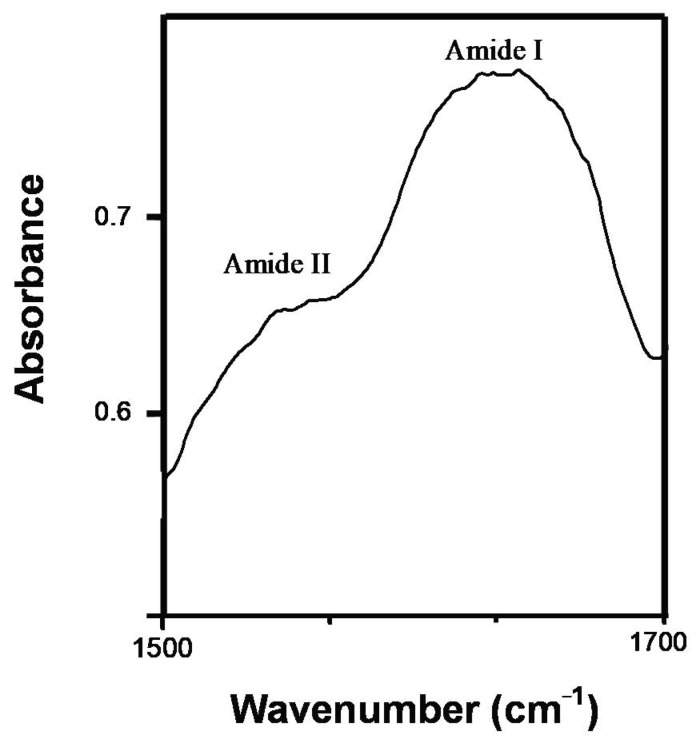
Typical FT-IR spectra from cooked sausage (type frankfurter) in the 1700–1500 cm^−1^.

The typical protein bands in the mid-infrared (IR) spectra are amide I (1700–1600 cm^−1^), amide II (1560–1510 cm^−1^) (Figure 2) and amide III (1300–1200 cm^−1^) which provide information on protein secondary structure (α-helix, β-sheet, turn, unordered). Modifications in the intensity and/or frequency of these infrared bands is indicative of protein structural modifications [29,30]. The amide I band, high intensity (Figure 2) due to carbonyl stretching vibration with a slight influence from C-N stretching and N-H bending vibrations, is the one most frequently employed to analyze the secondary structure of proteins. Proteins with α-helical conformation show robust amide I bands between 1657 and 1650 cm^−1^, whereas bands between 1640 and 1612 cm^−1^ are usually related with β-sheets.

**Figure 3:**
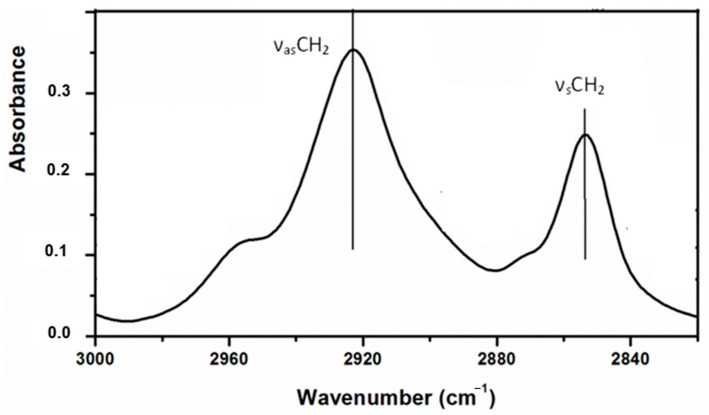
Representative FT-IR spectra in the 3000–2820 cm^−1^ region from cooked meat products (type frankfurter).

The mid-IR spectra of lipids encompass various bands in the 3000–1700 cm^−1^ region and overlapping bands in the 1500–700 cm^−1^ region. It is worth noting that in the elaboration of meat products with health-enhanced lipid content, infrared studies of the region between 3000–2800 cm^−1^ are characterized by two strong bands at 2925 and 2854 cm^−1^ (Figure 3) caused respectively from the asymmetric (ν_as_CH_2_) and symmetric (ν_s_CH_2_) stretching vibrations of the acyl CH_2_ groups of lipids [31,32,33]. Modifications in lipid chain order/disorder causing from protein–lipid interactions can alter the half-bandwidth of these bands [34].

#### 3.1.2. Raman Spectra

Raman spectroscopy offers data on protein structure, mostly by analysis of the amide I and III bands, which are associated with secondary structure. This spectroscopic technique also provides bands due to the environment of some side chains of proteins (aliphatic, tyrosine and tryptophan residues) and on the local conformations of disulfide bonds and methionine residues, all associated with a tertiary protein structure [27,29]. Fourier transform (FT) Raman spectrometers with 1064 nm excitation are commonly used to study the structure of these food products [13,27].

**Figure 4:**
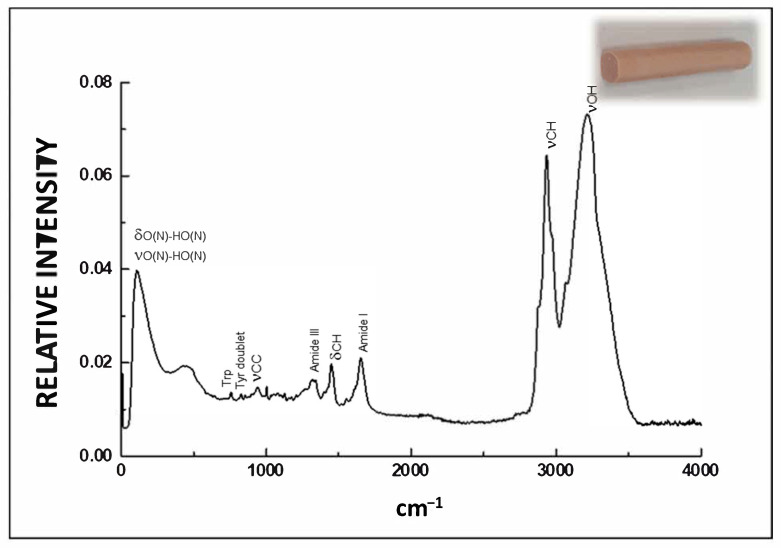
Typical Raman spectrum of a cooked sausage (type frankfurter) in the 0–4000 cm^−1^.

The principal Raman bands to establish the secondary structure of meat protein (α-helix, β-sheet, turn, unordered) are amide I (1650–1658 cm^−1^), which involves mostly C=O stretching, and amide III vibrational modes (1225–1350 cm^−1^) (Figure 4). Amide I, a strong band that involves mostly C=O stretching, is the most commonly used in the study of secondary protein structure. Most studies on the vibrational spectroscopy of proteins highlight the correlation between amide I band frequencies and protein secondary structure [27,29] proteins with great α-helical content, which display an amide I band centered about 1650–1658 cm^–1^ (Figure 4), while those with mainly β-sheet structures display the band between 1665 and 1680 cm^−1^, and a great content of unordered structure is relate to proteins with an amide I band centered at 1660–1665 cm^−1^. The spectral profile of the amide I band is employed in quantifying the secondary structure of proteins in terms of content of α-helix, β-sheet, turn and unordered using different methods mainly centered on frequency determinations at maximum intensity and half-bandwidth of the amide I band [27,29]. However, before this band can be analyzed, the water spectrum must first be correctly subtracted from the spectra. Additionally, other weaker bands can be noticed and analyzed in the Raman spectra. These correspond to the influence of peptide structure on the environment of some side chains such as those of aliphatic (δCHn), tyrosine (Tyr doublet) and tryptophan (Trp) residues (Figure 4), and on the local conformations of disulfide bonds and methionine residues associated to tertiary protein structure [35].

There are also several Raman bands allocated to lipids near 1750, 1660, 1470, 1443, 1306, and 1269 cm^−1^ allocated to the C=O stretching modes, C=C stretching modes, CH_2_ scissoring modes, CH_2_ twisting modes, and CH in plane deformation modes of lipids [6,7]. The unsaturation level of fat-containing food products can be assessed precisely by analyzing the C=C stretching band (1660 cm^−1^). Nevertheless, one of the most frequently used regions of the Raman spectra in the structural study of lipids in meat and meat products, especially those products with health-enhanced lipid content, is between 2800–3000 cm^−1^ (Figure 4) associated with changes in C-H stretching vibrations (νCH) [6,7]. A CH_3_ symmetric stretching band near 2897 cm^−1^, a CH_2_ asymmetric stretching band near 2930 cm^−1^ and a CH_2_ symmetric stretching motion near 2850 cm^−1^ are found in this region [29]. The symmetric and asymmetric vibrational modes of the CH_2_ and CH3 groups can offer information about interactions between hydrocarbon chains. The peak height intensity relations Iν_s_CH_2_/Iν_as_CH_2_ (I2850/I2890) and Iν_s_CH_3_/Iν_as_CH_3_ (I2935/I2890) offer valuable indices to measure lipid packing consequences and establish relative order/disorder of the intermolecular lipid chain [36,37].

Raman spectra also show a broad water band (3100 and 3500 cm^−1^) (Figure 4), associated with OH stretching motions [38,39], and a Raman band in the low-frequency range (below 600 cm^−1^) including the bending (δ) and stretching (ν) vibrations of the O(N)-H..O(N) units, due to interactions of hydrogen bonded to water and protein molecules [38,39].

## 4. Application of Vibrational Spectroscopy in Meat Products with Healthier Lipid Content

Once this basic knowledge about vibrational spectroscopic techniques has been established, their application on the development of EGs and OBAs and healthier meat products in which these structured lipids are incorporated will be described separately.

### 4.1. Infrared and Raman Spectroscopy to Study Lipid Material Based on Structured Lipids

Many researchers have studied the possibilities of vibrational spectroscopy to establish fat content and the fatty acid composition of animal fat (adipose tissue from beef, lamb, pork and chicken, etc.) [40,41,42,43] Raman spectroscopy has been used to predict PUFA, MUFA and SFA content, and the degree of unsaturation (IV) in melted fat and adipose tissue of pork [41]. Raman spectral regions between 775 and 1800 cm^−1^, and between 2600 and 3100 cm^−1^, were selected for regression models since these contain bands related to lipids. Results showed that Raman spectroscopy is an interesting technique to measure the fatty acid composition of pork adipose tissue with the benefit that this technique is non-invasive and measurements can be completed online [41]. Vibrational spectroscopy has also been used extensively to achieve structural information about oils [31,32,43,44,45], which are the basis of lipid materials. Based on the useful results obtained from the analysis of animal fat and oils, many studies were later conducted on lipid materials, especially structured lipids.

FT-IR has been employed to study conformational modifications of oils emulsified with proteins (α-lactalbumin and β-lactoglobulin) where these proteins are adsorbed in the emulsion formation process [46,47]. This process induces changes in their secondary structure [46,47]. The results showed the creation of an intermolecular, antiparallel β-sheet upon adsorption due to protein self-aggregation. Studies to obtain details on protein secondary structures of olive oil-in-water emulsions stabilized with various protein systems based on caseinate or soy protein have been conducted using FT-IR [48,49]. The relationship between emulsion structure and its physical properties was also evaluated. Protein secondary structures changed to more orderly protein backbones, mainly involving the α-helical structure upon creation of the olive oil-in-water emulsion. These structural properties could be associated with the firmer textural characteristics found in soy and caseinate emulsions. A better interpretation of the potential relationship between the structural and textural characteristics of olive oil-in-water emulsions could help in selecting the best emulsion components to obtain specific textural characteristics. In this connection, FT-IR and FT-Raman play an important role. Structured lipids such as emulsion gels (EGs) are an interesting possibility to structuring edible oils. These are described as emulsions with a gel-like network structure and solid-like mechanical characteristics [2,3], making them a good alternative to animal fat in meat products with a healthy lipid profile. It is relevant to note the function of structure and lipid phase interactions in the stability of EGs and their technological properties [4,5]. IR, especially attenuated total reflectance (ATR)-FTIR spectroscopy, has proven valuable insofar as it is a fast, non-destructive analytical method capable of offering analytical and structural information on various EGs. Molecular structures of polyphenol–soy EGs have been studied using this spectroscopic technique owing to their potential as release systems for bioactive compounds (polyphenols, MUFAs, PUFAs). Studies on polyphenol–soy EGs have shown that phenolic compounds seem to become confined in the emulsion matrix network, due to its textural properties, exhibiting lower gel strength than EGs without polyphenols [50]. ATR-FTIR spectroscopy has also been used to examine the structural characteristics of chia EGs. Analyses of the 3000–2500 cm^−1^ region indicate that the order/disorder of the oil lipid chain, associated to lipid interactions and droplet size in the emulsion gels, could be important in controlling their textural properties [4]. Raman spectroscopy has also been used to examine the lipid structure of different chia EGs and spectroscopic results, mainly significant differences were found in the area 2800–3000 and I2854/I2900, and indicated differences depending on these EG compositions in the lipid structure and interactions in terms of lipid acyl chain mobility (order/disorder). These lipid structural properties of chia oil EGs linked significantly with a particular textural behavior [9].

Polysaccharides, a structured lipid, can be employed either individually or in mixture to form a diversity of gel structures which may be appropriate for immobilizing oil droplets working as oil-bulking agents. Raman spectroscopy has also been employed to determine lipid and polysaccharide interactions in different polysaccharide gels elaborated for use as oil-bulking agents [5]. Raman spectroscopic results show that these structured lipids were stabilized by hydrogen bonding between oil carbonyl groups and water and/or carbohydrate molecules. This structural behavior may clarify the differences in textural characteristics. Structural studies of lipid materials (EGs and OBA) contribute to a better interpretation of the correlation between their technological properties and the structural characteristics of their ingredients. Understanding these correlations ultimately helps in selecting the most suitable materials.

This information is of particular interest given that these lipid materials can help to improve or maintain the quality of the food to which they are added. Particularly important in this regard is their use as animal fat replacers, for instance in the development of health-enhanced meat products without compromising the properties of the final product.

### 4.2. Infrared and Raman Spectroscopy to Examine Meat Products with Healthier Lipid Content

Vibrational spectroscopic techniques, particularly FT-Raman and FT-IR, provide information about the secondary structure of proteins and the structure of lipids in meat and meat products [6,13,27,28]. These spectroscopic techniques have been used to build models that can predict sensory and technological characteristics, and also to evaluate structural modifications appearing in proteins during processing [13,28,29]. Studies evaluating the feasibility of FT-IR spectroscopy in determining structural properties have focused on health-enhanced meat products reformulated with lipid material such as olive oil-in-water emulsions stabilized with casein or soy protein to replace animal fat [51]. The results of these studies suggest that this spectroscopic technique offers relevant information about changes in protein secondary structure caused by changes in the amide I band profile. Moreover, changes in the 3000–2500 cm^−1^, particularly the half-bandwidth in the 2922 cm^−1^ band, has suggested modifications in lipid chain order or lipid–protein interactions. These results underscore the relevance of the stabilizing system employed in the preparation of oil-in-water emulsions as it impacts both the structural and technological characteristics of the reformulated meat products.

**Figure 5:**
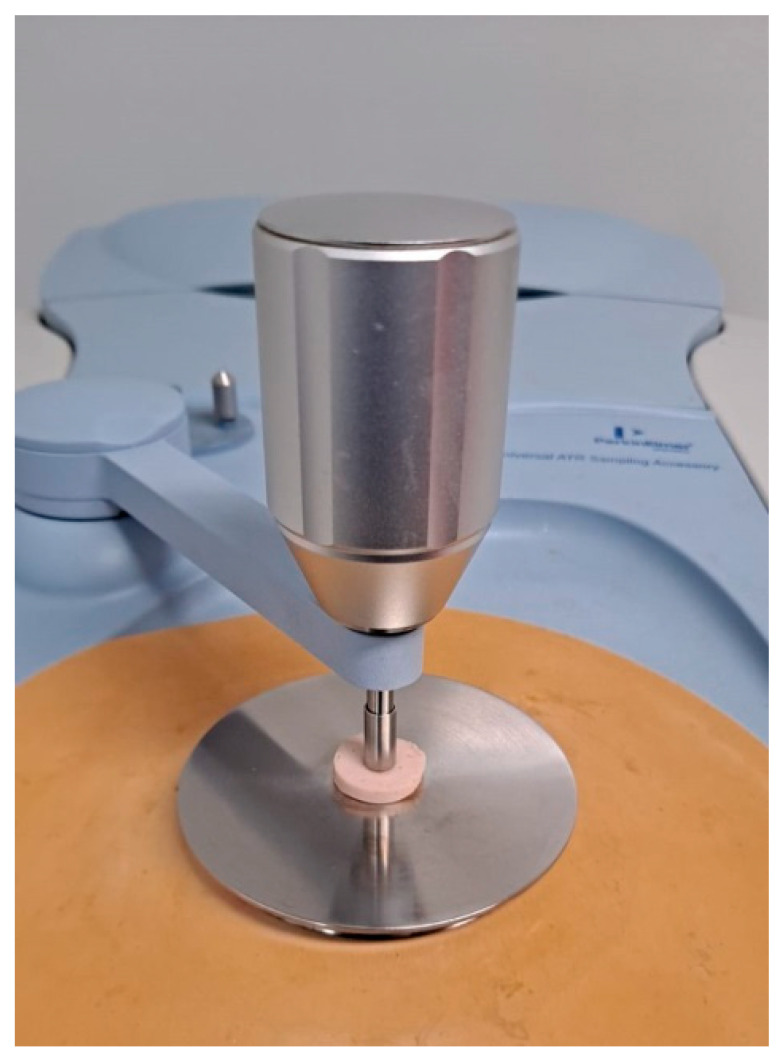
Analysis of frankfurters reformulated with structured lipids based on polyphenol-EGs as animal fat replacers using FT-IR with an ATR device.

Lipid materials based on structured lipids such as chia EGs have been applied as animal fat substitutes to develop frankfurters with a health-enhanced lipid profile. In this case, ATR-FTIR spectroscopy was used to study how replacing animal fat with chia EGs affects the structural characteristics of lipids [52]. Results from the 3000–2500 cm^−1^ region showed that frankfurter reformulated with chia emulsion gels implicate more lipid–protein interactions which correlated significantly with processing loss (mainly loss of water and fat) and the textural behavior of these samples [52]. ATR-FTIR spectroscopy was also used to study health-enhanced frankfurters reformulated with structured lipids based on polyphenol-EGs as animal fat replacers (Figure 5). The spectroscopic result of the acyl chain region (between 2950 and 2830 cm^−1^) of the ATR-FTIR spectrum presented greater (*p* < 0.05) inter- and intramolecular lipid disorder irrespective of the presence of polyphenol in the EG [53].

FT-Raman spectroscopy was also employed to study the structural properties of health-enhanced frankfurters reformulated with structured lipids such as oil-bulking agents to replace fat [6]. Analysis of the amide I band furnished valuable information on secondary protein structures regarding enrichment of β-sheet structures in these frankfurters reformulated with oil-bulking agents [6]. The high content of β-sheet structures in these health-enhanced frankfurters indicate the creation of a denser network, resulting in increased hardness. Regarding lipid structure, analysis of the 2800–300 cm^−1^ regions and intensity ratios Iν_s_CH_2_/Iν_as_CH_2_ (I2850/I2890) and Iν_s_CH_3_/Iν_as_CH_3_ (I2935/I2890) revealed that the addition of oil-bulking agents as animal fat replacers in these health-enhanced frankfurters increased lipid acyl chain disorder and implicated more lipid−protein interactions [6].

## 5. Conclusions

The notion of improving the lipid component in meat products has been gaining attention in recent years giving rise to the development of new reformulation strategies to obtain healthy meat products and optimal technological properties. New procedures to create lipid materials based on structured lipids, such as EGs and OBAs, are especially interesting owing to their solid-like properties and the fact that they confer valuable nutritional and technological properties to meat products into which these materials have been added. EGs and OBAs have been employed successfully as animal fat replacers in the development of different meat products (sausages, hamburgers, etc.) with healthier lipid contents (qualitative and quantitative).

Application of vibrational spectroscopy (infrared (IR) and Raman spectroscopy) has significant possibilities and an increasing range of applications in the analysis of structured lipids (mainly EGs and OBAs) and meat products into which these lipid materials have been incorporated for the purpose of achieving healthier lipid contents. The use of vibrational spectroscopic techniques for both lipid materials and health-enhanced meat products have provided us with a greater insight into how the structure of their main components (proteins and lipids) impacts technological properties, mainly texture. This information can be helpful in choosing the most appropriate compounds for lipid materials used as animal fat replacers to obtain specific technological behaviors. Structural information and its correlation with certain technological properties could help in the selection of the most optimal lipid material and other compounds to obtain certain properties in health-enhanced meat products into which they are included.

## Data Availability

The data presented in this study are available on request from the authors
